# Antimicrobial Characterization of Site-Directed Mutagenesis of Porcine Beta Defensin 2

**DOI:** 10.1371/journal.pone.0118170

**Published:** 2015-02-26

**Authors:** Xian-xian Huang, Chun-yu Gao, Qing-jun Zhao, Chun-li Li

**Affiliations:** Department of Animal and Veterinary Science, Henan Agricultural University, Zhengzhou, Henan Province, The People’s Republic of China; University of Central Florida College of Medicine, UNITED STATES

## Abstract

Porcine β defensin 2 (pBD2) is a small, cationic and amphiphilic antimicrobial peptide. It has broad antimicrobial activities against bacteria and plays an important role in host defense. In order to enhance its antimicrobial activity and better understand the effect of positively charged residues on its activity, we substituted eight amino acid residues with arginine or lysine respectively. All mutants were cloned and expressed in BL21 (DE3) plysS and the mutant proteins were then purified. These mutant versions had higher positive charges but similar structural configurations compared to the wild-type pBD2. Moreover, these mutant proteins showed different antimicrobial activities against *E. coli* and *S. aureus*. The mutant I4R of pBD2 had the highest antimicrobial activity. In addition, all the mutants showed low hemolytic activities. Our results indicated that the positively charged residues were not the only factor that influenced antimicrobial activity, but other factors such as distribution of these residues on the surface of defensins might also contribute to their antimicrobial potency.

## Introduction

Defensins are a group of small, cationic, and cysteine-rich antibacterial peptides. They have activities against pathogens, which makes them ideal candidates for novel therapeutic drugs [1–8]. Mammalian defensins are classified into α, β and θ defensins based on the connectivity of three disulfide bridges of cysteine residues [3]. β defensins are the oldest one in the evolution [7] and are made up of 30–45 amino acid residues, with 5–12 positively charged residues such as lysine and arginine. The typical β defensins have a very stable structure composed of one alpha helix and three beta sheets [1–2].

β defensins are the only mammalian defensins found in pigs. Except for porcine β defensin 1 (pBD1), all other porcine β defensins are discovered by genomic sequence analysis. Relatively little work have been done on porcine defensins and they have not yet been isolated from natural sources [7, 9–10]. pBD1, the first member of porcine β defensin, has a strong inhibitory activity against gram-negative bacteria [11–12]. On the contrary, porcine β defensin 2 (pBD2) shows strong antibacterial activity against both gram-negative and gram-positive bacteria, including multi-resistant bacteria. Therefore, pBD2 has a great potential to be a good candidate to replace antibiotics [13–14]. The activities of the other 9 kinds of porcine β defensins have not been reported yet.

The mechanism of defensins against bacteria has been studied for years. However, there are still controversies about it. Currently, it has been established that the primary mechanism for defensins to target bacteria is by interaction of the positively charged residues of denfensins with the negatively charged components of bacterial cell membrane. Many studies have hypothesized that the positive charge or isoelectric point (pI) of the defensin proteins is the major factor influencing their antimicrobial activity [14–16]. The positively charged residues interact with the negatively charged microbial components such as lipopolysaccharide or phospholipids to disrupt the cell membrane, thereby causing bacterial death directly or increasing the bacterial membrane permeability, or targeting the internal structures such as DNA or RNA by entering into the bacteria to kill the bacteria [5, 16–18]. Though some reported that the structure was not necessary for their antimicrobial activities, it could explain the stability of defensins to defend against digestion by bacterial enzymes [2, 5, 6, 18–19].

In order to enhance the antimicrobial activity and better understand the role of the positive charges of defensins on the antimicrobial efficacy, different pBD2 variants were constructed using site-directed mutagenesis. The resulting altered recombinant peptides contained more positive charges compare to the original peptide sequence without changing the secondary structure. After expression in *E. coli*, these recombinant mutated proteins were purified and subjected to antibacterial and hemolytic assays.

## Materials and Methods

### Bacterial Strains

*E.coli* TG1 and BL21 (DE3) plysS were used for cloning and expression respectively (maintained in our laboratory). The *E. coli* ATCC 25922 and *S. aureus* ATCC 25923 were purchased from the Beijing Ordinary Microbiology Strain Store Center, Beijing, China.

### Selection of point mutations of pBD2

Residues were selected for exchange by mutagenesis based on the analysis of physicochemical properties and secondary structures of different single-point mutated pBD2. The physicochemical properties were predicted using online software http://us.expasy.org/tools and other software reported before [20]. The secondary structures of pBD2 and its mutants were predicted at the website: http://swissmodel.expasy.org/.

### Construction of the expression vectors

Different site-directed genes were obtained by PCR using the primary pBD2 gene as the template. The primers with different mutational sites were designed and synthesized by Shanghai Sangon Biotechnology Co., Ltd (Table 1). *Nco* I /*Hind* III endonuclease sites were introduced to their 5’ and 3’ end respectively.

**Table 1:** Primer sequences for site-directed mutagenesis.

Primer Name	Primer Sequence
pBD2-F	5’- GCGCCATGGCTGACCACTACATATGTGCCAAG-3’
pBD2-R	5’- GCGAAGCTT TTATTAGCGGATGCA-3’
D1K-F	5’- GCGCCATGGCT**AAA**CACTACATATGTGCCAAG-3’
I4R-F	5’- GCGCCATGGCTGACCACTAC**CGT**TGTGCCAAG-3’
S15R-F	5’-GCGCCATGGCTGACCACTACATATGTGCCAAGAAAGGGGGGACCTGCAACTTC**CGT**-3’
L19R-F	5’-GCGCCATGGCTGACCACTACATATGTGCCAAGAAAGGGGGGACCTGCAACTTCTCCCCCTGCCCG**CGT**-3’
I23K-R	5’-GCGAAGCTT TTATTAGCGGATGCAGCACTTGGCCTTGCCACTGTAACAGGTCCCTTC**TTT**-3’
E24R-R	5’-GCGAAGCTT TTATTAGCGGATGCAGCACTTGGCCTTGCCACTGTAACAGGTCCC**ACG**AAT-3’
T26K-R	5’-GCGAAGCTT TTATTAGCGGATGCAGCACTTGGCCTTGCCACTGTAACA**TTT**CCCTTC-3’
S29R-R	5’- GCGAAGCTT TTATTAGCGGATGCAGCACTTGGCCTTGCC**ACG**GTA-3’

Note: *Nco*I and *Hind*III restriction sites were marked in single underline; the mutated sites were marked in bold and double underline; terminal codons were marked in curved underline.

The PCR products were cloned into pMD19-T vectors (Takara, Japan), and transferred into *E. coli* TG1 cells respectively. After identification by PCR, double enzyme digestion and DNA sequencing, the positive vectors were digested by *Nco* I / *Hin*d III. Different pBD2 gene variants were inserted into pET-30a vectors (Novagen) respectively for generation of proteins containing a N-terminal His-tag used for protein purification. Finally, the positive vectors with different site-directed mutated genes were transformed into *E. coli* BL21 (DE3) PlysS for expression respectively.

### Expression and purification of the proteins

The different strains *E. coli* BL21 (DE3) PlysS, harboring different expression vectors, were induced by 1mM Isopropylthio-D-galactoside (IPTG) until the optical density (OD_600_) reached up to 0.6~0.8 respectively. The different cells were harvested respectively and purified according to the protocol described previously [21]. Generally, cells were disrupted by sonication using an Ultrasonic Cell Disruption System (Scientz Ltd, China). The target protein was purified by Ni-NTA agarose column. The purified protein was dialyzed overnight against 20 mM phosphate buffer (pH 7.4) and concentrated by polyethylene glycol 8000. The protein concentrations were quantified by UV absorption at 280 nm and 260nm using a spectrophotometer (2800 UV/VIS, UNICO instruments Co. Ltd., China).

### Antibacterial activity assay

The antimicrobial activities were evaluated by the turbidimetric method [22]. The bacteria were diluted to 10^5^~10^6^ CFU/ml in LB medium, 100 μL of diluted bacteria was then mixed with 100 μL of individual mutated pBD2 defensins and plated in a 96-well plate (final concentrations were 0~80 μg/ml). The growth of bacteria was determined as increase of the optical density at 630 nm (reference 405 nm) at different time points (8h, 16h and 24h) using a microplate reader (Stat Fax 2100, Awareness Technology Inc. USA). Survival percentage was defined as the ratio of optical density of bacteria with defensin to that without defensin. All assays were performed in triplicates. All values are expressed as mean ± standard deviation (SD).

### Hemolytic activity assay

The release of hemoglobin from porcine erythrocytes was used as a measure for the hemolytic activity of defensin as described before [13–14, 23]. 1mL fresh porcine blood was centrifuged at 1800 g for 10 min for collecting erythrocytes in the presence of heparin to prevent coagulation. The pellet was washed three times and re-suspended in saline solution at the proportion of 1:9. Subsequently, 100 μL erythrocyte solution was mixed with 100 μL of individual mutated defensins (final concentrations were 0~80 μg/ml) in polypropylene 96-well microtiter plates. After incubation at 37°C for 1 h, the plate was centrifuged at 1800 g for 5 min. 100 μL supernatant was transferred to a new 96-well plate separately and the absorbance was measured at 450 nm (reference 630 nm) using a microplate reader. The hemolysis ratio was calculated by comparison with the control samples containing no peptide or 1% Trition-100 [24–25].

### Modeling

The tertiary structures of pBD2 and its mutants were predicted based on the homology modeling of human β defensin 1 (hBD1) and its analogues because their amino acid sequences had more than 60% identity. The sequences were subjected to a homology search at http://swissmodel.expasy.org/ respectively, and the most similar sequence was selected as a template to build a model for each sequence. Finally, the pBD2 and its mutants were further modeled using the software Swiss PBD Viewer (version 4.1.0).

### Statistical analysis

Analysis of variance (ANOVA) was implemented by SPSS 17.0. Means were compared by LSD or Dunnett. Statistical significance was defined at *P* < 0.05.

## Results

### Selection of Point Mutations of pBD2

Mature pBD2 contains 37 amino acids (GenBank accession No. AY506573.1), among them there are 2 acidic and 7 basic amino acids. In order to increase the net positive charges, we selected acidic or neutral amino acids to be replaced by basic amino acids. The selection was based on analysis of the physicochemical properties and secondary structures of each single-point mutated pBD2. The selected mutated residues were predicted to retain the secondary structures, and all glycine, cysteine and proline residues were excluded from mutagenesis. Altogether, eight residues were substituted for arginine or lysine, which were D1K, I4R, S15R, L19R, I23K, E24R, S29R and A32K and will be referred to as mpBD2-1~8 successively. In essence, D1K would mean that the aspartic acid (D) residue in the first position of the sequence was replaced by lysine (K), and so on. It was reported that hBD1 had enhanced activities when lysine residues were substituted for arginine residues [8]. Therefore, arginine was the preferred choice for substitution while lysine was the second choice whenever arginine substitution caused instability according to the predicted instability index. The mutations D1R, I23R and A32R were unstable and therefore excluded from this study (data not shown). The physicochemical properties of all the selected mutants are listed in Table 2. Such substitutions increased positive charges and hydrophilicity of the peptides. In addition, all the polypeptides were stable and thermo-stable according to the instability and aliphatic indexes.

**Table 2:** Parameter prediction of site-directed mutagenesis.

Peptide	Net positiveCharge	pI	relative hydrophobic moment (uHrel)	mean hydrophobic moment(uH)	mean hydrophobicity(H)	Grand average of hydropathicity (GRAVY)	Instability index	Aliphatic index
PBD2	5	8.89	0.06	0.4	-2.04	-0.289	38.62	47.57
D1K(mpBD1)	7	9.27	0.07	0.44	-2.08	-0.300	38.62	47.57
I4R(mpBD2)	6	9.10	0.12	0.79	-2.54	-0.532	34.05	37.03
S15R(mpBD3)	6	9.10	0.08	0.53	-2.19	-0.389	31.95	47.57
L19R(mpBD4)	6	9.10	0.14	0.93	-2.57	-0.514	36.58	37.03
I23K(mpBD5)	6	9.08	0.13	0.84	-2.54	-0.516	26.74	37.03
E24R(mpBD6)	7	9.30	0.05	0.36	-2.06	-0.316	24.45	47.57
S29R(mpBD7)	6	9.10	0.07	0.46	-2.19	-0.389	31.75	47.57
A32K(mpBD8)	6	9.08	0.04	0.27	-2.28	-0.443	38.62	44.86

**Fig 1:**
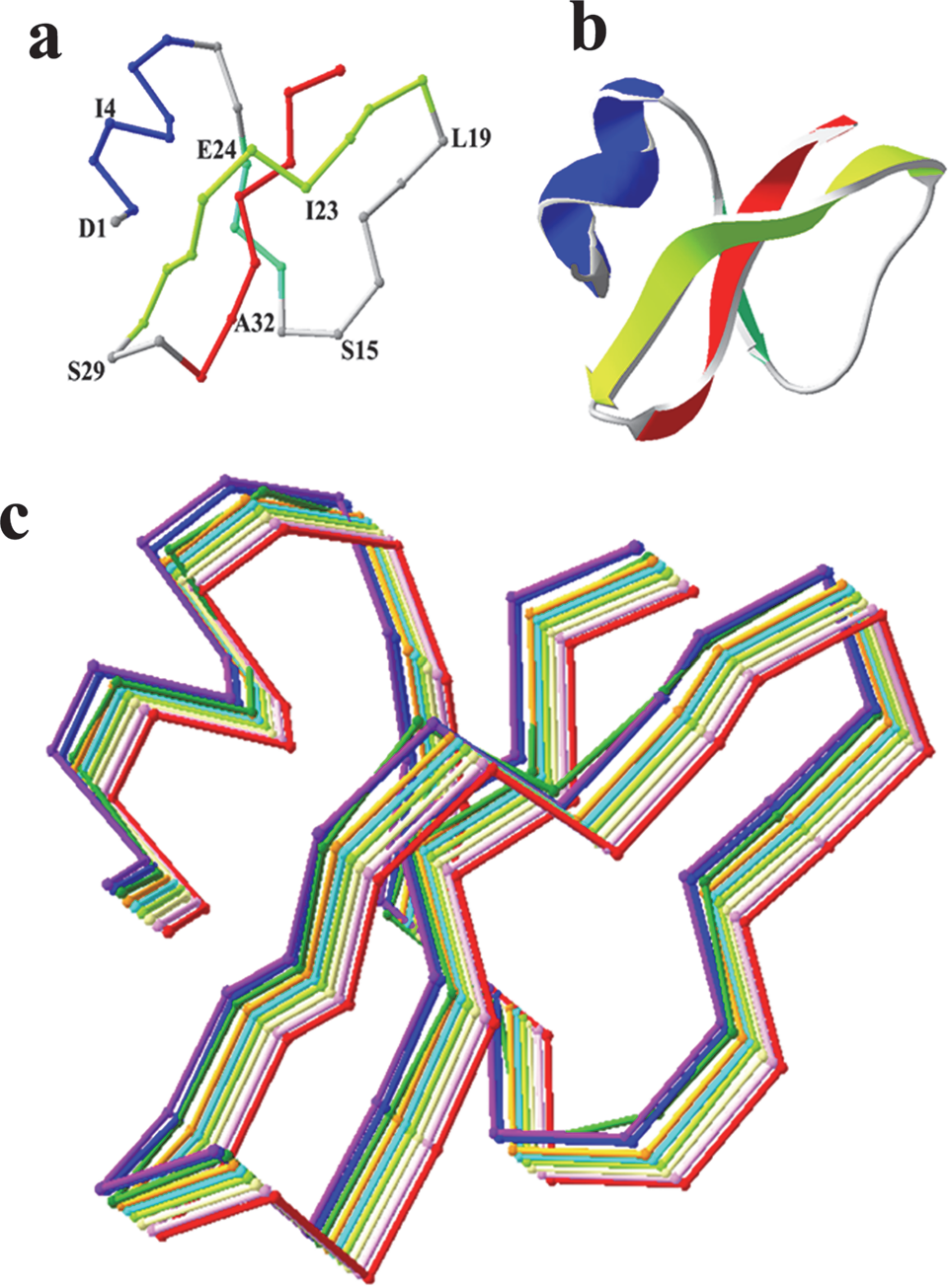
Secondary structural alignment of pBD2 and its mutants. (a) Backbone as Cα trace of pBD2 with location of mutated residues. (b) Secondary structure of pBD2. Color in a and b represented the residues for different structures: Blue for α helix; cyan, green and red for the first, second and third beta sheets respectively. (c) Structural alignment of backbones of pBD2 and its mutants. Color represented the backbone Cα trace of pBD2 and different mutants. Red for pBD2.pink, yellow, green, cyan, orange, dark green, blue and violet for D1K, I4R, S15R, L19R, I23K, E24R, S29R and A32K respectively.

The amino acid sequences of pBD2 and hBD1 had 60% identity, and the mutants of pBD2 had 61.11%, 63.89% or 66.67% identity to hBD1 and its analogue. The structure of hBD1 is composed of one alpha helix (His^2^-Ser^7^) and three beta sheets (Gln^11^-Leu ^13^, Ile^23^-Cys^27^ and Lys^32^-Lys^35^) [18–19]. All the mutants and pBD2 had a similar predicted secondary structure with one alpha helix (His^2^-Lys^8^) and three beta sheets (Thr^11^-Asn^13^, Phe^20^-Tyr^28^ and Lys^31^-Arg^37^) (Fig. 1). None of the mutant proteins had significant change in their secondary structure, which implied that their activities were only associated with the changes in their respective side chains.

### Construction of the expression vectors of different site-directed mutagenesis

The different site-directed mutant genes were obtained by PCR and ligated with pMD19-T vectors respectively. Positive recombinant plasmids T-m*pBD2-1~8* were confirmed by PCR and double enzyme digestion with *Nco* I and *Hin*d III. The sequencing results confirmed that the cloned sequences were consistent with the design. After enzyme digestion, the mutated genes were cloned into expression vector pET30a between *Nco* I and *Hind* III resulting in recombinant plasmids pET-*mpBD2-1~8*. The sequencing results showed that all the recombinant plasmids were successfully constructed.

### Expression and purification of different mpBD2-1~8

**Fig 2:**
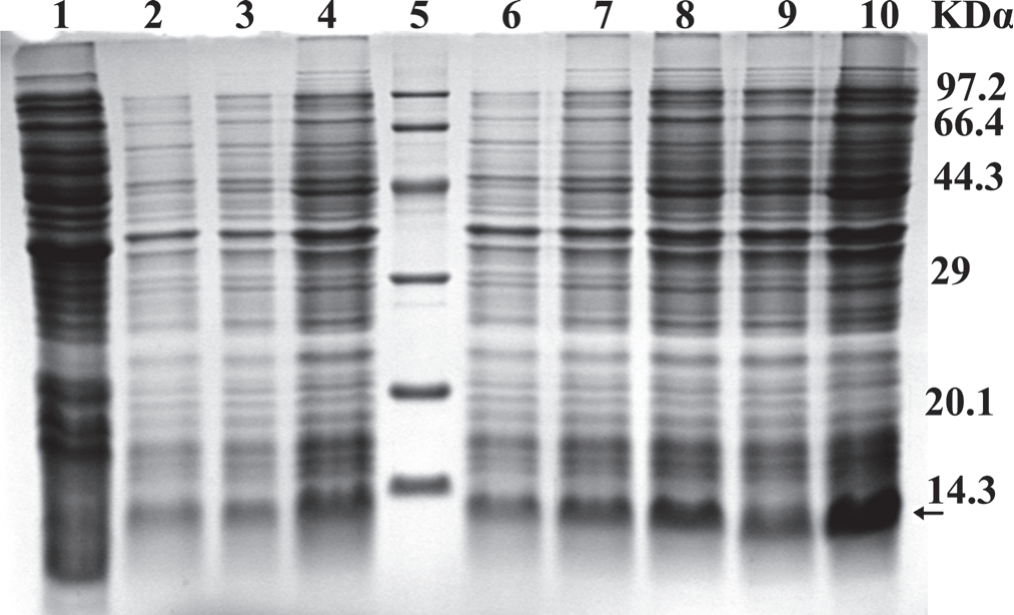
SDS-PAGE analysis of expressed recombinant mpBD2-1~8. Lane 1 showed the BL-PET30 as negative control; Lane 2~4 and lane 6~10 showed different recombinant mpBD2-1~8 expressed in *E. coli* respectively; Lane 5 indicated the protein marker; The arrow indicated the interested protein.

**Fig 3:**
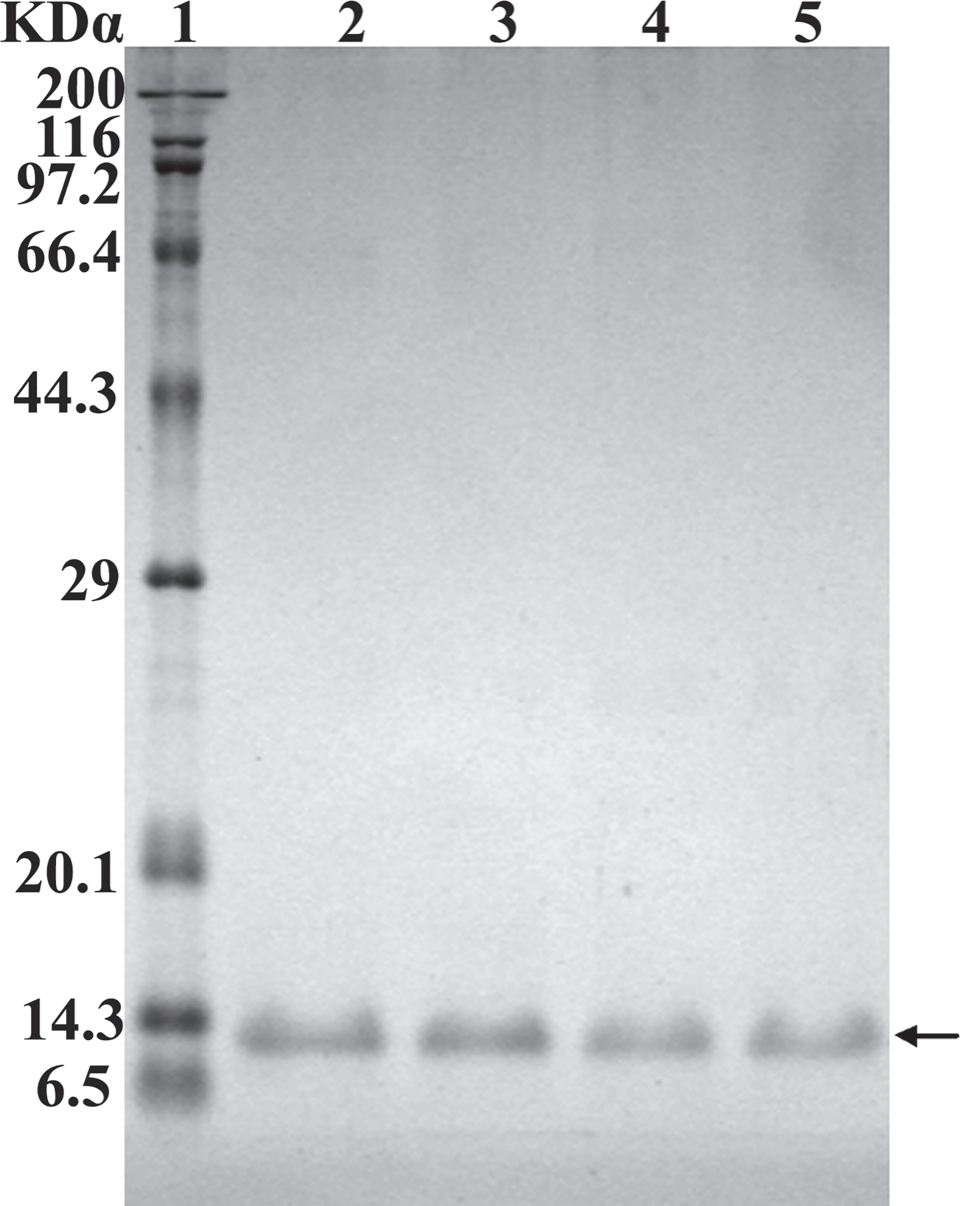
SDS-PAGE analysis of purified recombinant mpBD2 by affinity column. Lane 1 indicated protein marker; Lane 2~5 indicated different fractions from Ni-NTA agarose column with the elution buffer. The arrow indicated the interested proteins. The proteins were stained by coomassie brilliant blue.

The strains *E. coli* BL21 (DE3) plysS, harboring different expression vectors (mpBD2-1~8), were induced with 1mM IPTG and the recombinant fusion proteins were successfully expressed (Fig. 2). The target proteins accounted for 12.8%~23.14% of total strain proteins according to the analysis by Gel-Pro Analyzer (4.0) software. After purification by Ni-NTA agarose column, protein yield with high purity (>95%) was obtained. Fig. 3 was an example of purified mpBD2-1. The purity of other mutant proteins was similar (data not shown). The molecular weight of these fusion proteins was about 10kDα according to analysis by Gel-Pro Analyzer (4.0), close to the theoretical value of the proteins.

### Antibacterial activity of mpBD2-1~8

**Fig 4:**
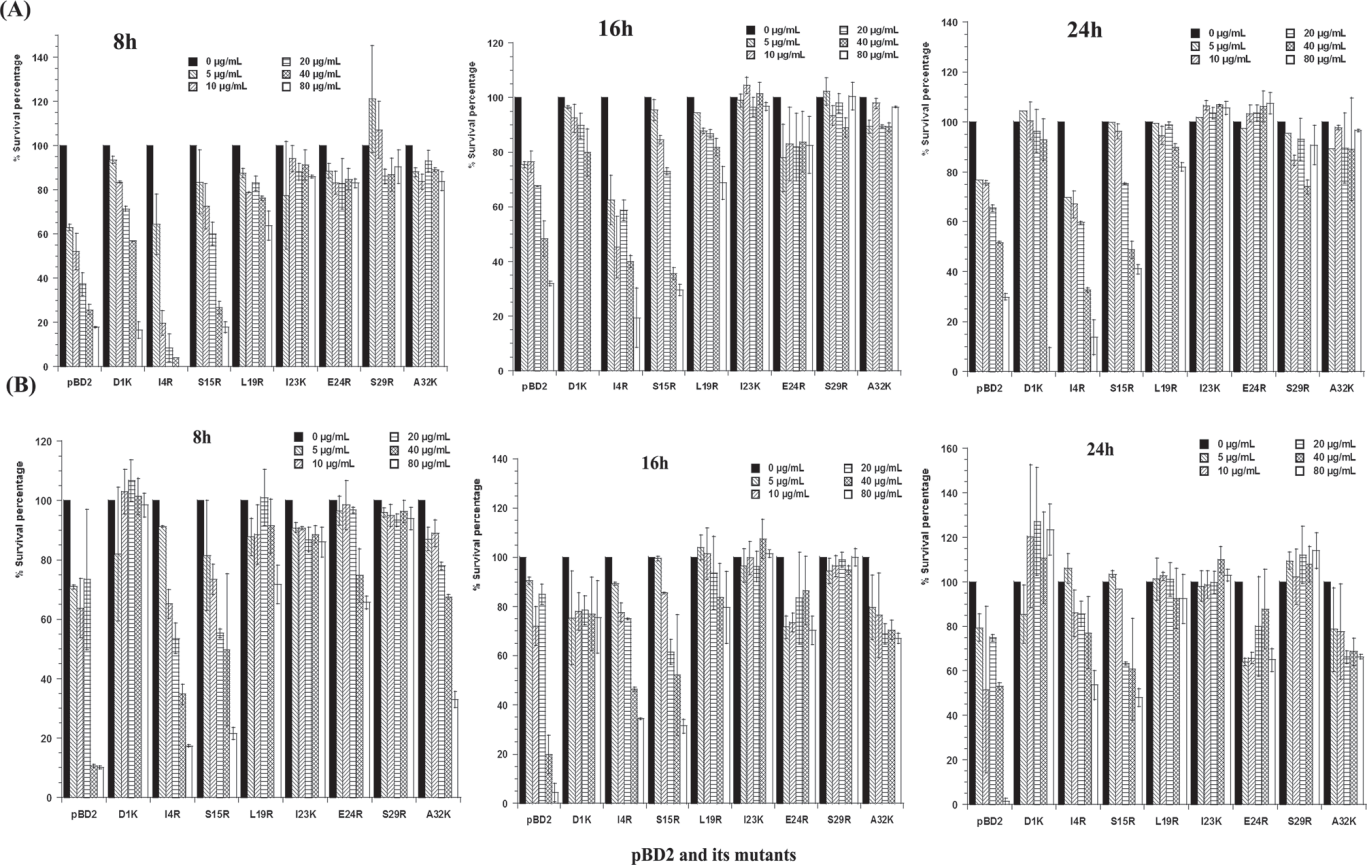
Antimicrobial activities of recombinant pBD2 and its mutants against *E. coli* (A) and *S. aureu*s (B) at different time points. The diluted bacteria were cultivated with different defensins (0~80 μg/mL) for 24 h. The optical density at 630 nm (reference 405 nm) was measured at different time points. Survival percentage was accounted as the ratio of optical density of bacteria with defensin to that without defensin. All assays were carried out in triplicates.

The activities of mpBD2-1~8 against *E. coli and S. aureus* were shown in Fig. 4. All the mutants showed antimicrobial activities. The anti-*E. coli* activities of mpBD2-1~8 were shown in Fig. 4A. Only one mutant, I4R, had higher activity than recombinant pBD2 at all the time points (*P* < 0.05). S15R had a similar activity with recombinant pBD2, while other 6 mutants showed lower activities than recombinant pBD2 at all time points (*P* < 0.05). The activities increased with concentrations at the selected time points for all the mutants and recombinant pBD2 (*P* < 0.05), while the activities were lower with time (*P* < 0.05).

The anti-*S.aureus* activities of mpBD2-1~8 were shown in Fig. 4B. None of the mutants had higher activities than recombinant pBD2. I4R and S15R had lower anti-*S. aureus* activities than recombinant pBD2 at all time points, but did not show significant difference (*P* > 0.05). At 16 h and 24 h, the anti-*S. aureus* activities of A32K, I4R and S15R were not significantly different from recombinant pBD2 (*P* > 0.05). E24R besides three mutants above (I4R, S15R and A32K) showed lower anti-*S. aureus* activities than recombinant pBD2 at 24 h without significant difference (*P* > 0.05). The other mutants had lower activities than recombinant pBD2 with significant difference (*P* < 0.05). The anti-*S.aureus* activities of defensins increased with concentrations at 8 h and 16 h (*P* < 0.05). Significant differences were observed between all the different concentrations with the control (*P* < 0.05). The anti-*S.aureus* activities reduced with time (*P* < 0.05).

Taken together, I4R was the best mutant for inhibiting *E. coli* and *S. aureus*. S15R had similar activities with recombinant pBD2.

### Hemolytic activity assay

Different concentrations of mutant pBD2 protein (100 μL) were separately incubated with erythrocytes (100 μL) at 37°C for 1 h. Less than 10% hemolysis ratio was found at all different concentrations of mpBD2-1~8 (data not shown). No significant differences were observed among different concentrations or among different mutants (*P* > 0.05). These results were consistent with previous reports by Veldhuizen *et al* and ours [13, 26], which indicated that the mpBD2-1~8 had very low hemolytic activities.

### Modeling

**Fig 5:**
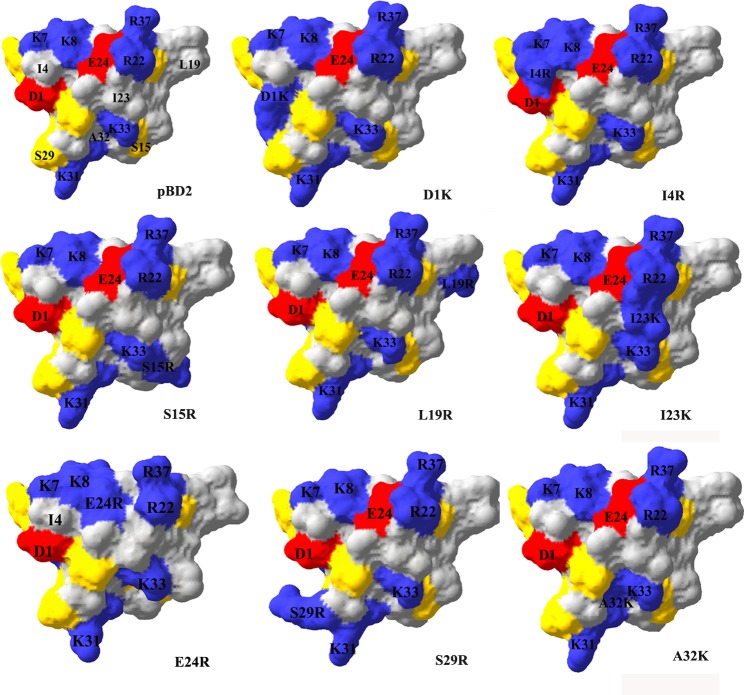
Molecular surfaces of pBD2 and its mutants. Molecular surface of pBD2 and its mutants were analyzed by Swiss PBD Viewer (version 4.1.0). Different color represented different residues type: blue for positively charged residues (K and R), red for negatively charged residues (D and E), yellow for polar residues (S, Y, T and N) and gray for non-polar residues (I, C, A, G, F, P and L).

The amino acid sequences of pBD2 and its mutants had more than 60% identity with hBD1 and its analogue. Therefore, it was credible for the homology modeling to be based on hBD1 and its analogue. The results showed that pBD2 and its mutants had similar tertiary structures, and their molecular surfaces were shown in Fig. 5. The different mutated peptides had different distribution of positive charges.

## Discussion

In this report, eight amino acids of pBD2 were selected for substitution by arginine or lysine based on rational analysis. It was hypothesized that the major factor defining the antimicrobial activities of defensins was their cationicity or isoelectric point (pI) values [14–16, 18, 27]. Indeed, the antimicrobial activities of defensins increased with their net charges [14–16, 18, 27–30]. Harder et al reported that human beta defensin 3 (hBD3) had the strongest activity among hBD1, 2 and 3, because it had the most net positive charges (hBD1, 2 and 3 has +5, +6, and +11 net positive charges respectively) [28–29]. Consistently, the activities of defensins reduced when their basic amino acids were replaced by acidic amino acids or other amino acids [18, 30], while the activities increased when the acidic or neutral amino acids were changed into basic amino acids [16, 18].

It was reported that hBD1 had stronger activities when four lysine residues were substituted for arginine residues [8]. Therefore, arginine was the preferred choice. By substitutions, the mutated pBD2 had more positive charges, higher relative hydrophobic moments and mean hydrophobic moments except for A32K (Table 2). Moreover, the amphiphilicity of these peptides increased, which was also important for activities of defensins [2, 31].

The defensins without the correct secondary structure also had high antimicrobial activities [16, 26, 32–34], which indicated that the structure was not necessary for their activities. However, the structure was important for their stability against digestion by enzymes [2, 5, 6, 9,18]. pBD2 and all the mutants had similar secondary structures and indicated typical structures of beta defensin shapes [35]. Thus, the results obtained on their antimicrobial activities might be not directly related to significant changes in their structures.

The mutated recombinant pBD2 molecules differed in their antimicrobial effect on gram-negative *E. coli* 29522 and gram-positive *S. aureus* 29523. Some mutants had higher or similar antimicrobial activities compared to the recombinant pBD2, while others had lower antimicrobial activities. Surprisingly, D1K and E24R had the highest net positive charge, but they did not have the highest activities. Paziger et al reported that the aspartic acid (Asp) residue was important for stability of hBD1 and hBD2 (equivalent to Asp^1^ in pBD2) [18, 36], and thus it may contribute to high antimicrobial activities of these proteins [18, 36]. It is possible that unstable structures could underlie lower antimicrobial activities of D1K. The similar reason may also explain the low antimicrobial activities of E24R, I23K and A32K, all of which are located in the beta sheets and is critical for pBD2 folding. These results also imply that the net positive charge is not the only factor determining the antimicrobial activities of defensins. Moreover, the stability of the molecule structure is important, which in turn is dependent on the arrangement of the amino acids. Previous studies suggested that the distribution of positively charged residues affected the antimicrobial activities of defensins [24, 31, 37]. In this study, all the mutated pBD2 had more positively charged amino acids (Fig. 5), but only I4R had higher antimicrobial activities. It was found that five out of eleven pBDs and eleven out of twenty-three hBDs had basic amino acids (lys or arg) in the same location (equivalent to R^4^in I4R) [38–39], suggesting that the basic amino acids were conserved during evolution. Along the same lines, some reported that N terminal and C terminal of defensins, which usually contains positively charged residues, were important for antimicrobial activities of hBD1 and hBD3 [16, 18]. Further studies are needed to explore the detailed mechanisms underlying positively charged residues in antimicrobial activities of pBD2.

Interestingly, our results showed that most mutants with arginine substitutions had no significant differences compared to the recombinant pBD2 in their anti-*S. aureus* activities. However, some have reported that the antibacterial effect caused by arginine substitutions on human α defensin 1 was much more pronounced on *Staphylococcus aureus* than on *Escherichia coli* [8]. In general, the antimicrobial activities of all the mutants and recombinant pBD2 increased with concentrations. These mutants with higher antimicrobial activities could inhibit the bacteria for a prolonged time, as long as 24 h, indicating that the action was long-lasting and ideal for their application in clinical practice. In our study, we found that I4R showed the highest activity against *E. coli* and *S. aureus*, perhaps owing to its high net positive charge, stability and charge density in its N terminal.

Recombinant pBD2 and its mutants had low hemolytic activities. These peptides had little cytotoxicity to porcine erythrocytes, consistent with previously reported studies [13, 26]. The low hemolytic activities offer advantages for its application in practice.

In conclusion, site-directed mutant pBD2 proteins showed different antimicrobial activities against *E. coli* and *S. aureus*. The most pronounced antimicrobial activities were found with mutant I4R. All the mutants had similar secondary and tertiary structures with the wild-type pBD2. Our results suggest that antimicrobial activity of pBD2 does not purely rely on the positively charged residues but also depends on other factors, including their arrangement on the molecule surface and their effect on molecule and structure stability.
